# Observed Versus Expected Distribution of Patient Self-Reported Race and Ethnicity in Quality Improvement Review Processes at a Single Safety Net Academic Institution

**DOI:** 10.7759/cureus.36090

**Published:** 2023-03-13

**Authors:** Delisa Quayson, Meredith Alston, Stefka Fabbri

**Affiliations:** 1 Obstetrics and Gynecology, University of Colorado Anschutz Medical Campus, Aurora, USA; 2 Obstetrics and Gynecology, Saint Joseph Hospital, Intermountain Health, Denver, USA; 3 Obstetrics and Gynecology, Denver Health and Hospitals, Denver, USA

**Keywords:** severe maternal morbidity, patient safety and quality improvement, safety intelligence, maternal race, maternal ethnicity, health inequity, health equity, health disparity

## Abstract

Background

While there is a plethora of evidence describing racial and ethnic disparities in obstetric care and outcomes, little has been published evaluating potential inequities in departmental Patient Safety and Quality Improvement (PSQI) processes.

Objective

The study aims to describe the distribution of patient-reported race or ethnicity for safety events at a single safety net teaching hospital. We hypothesized that the observed versus expected case distribution for each racial or ethnic group would be similar, signifying proportional representation in the PSQI reporting and review process.

Study design

We performed a cross-sectional study including all Safety Intelligence (SI) events filed on obstetric and gynecologic patients and all cases reviewed at monthly PSQI multidisciplinary departmental meetings from May 2016 to December 2021. We compared the distribution of patients' self-reported race or ethnicity as documented in the medical record to our patient population's expected race or ethnicity distribution based on historical institutional data.

Results

Two thousand and five SI events were filed on obstetric and gynecologic patients. Of those, 411 cases were selected for review by the departmental multidisciplinary PSQI committee, which meets once monthly. Of the 411 cases reviewed by the PSQI committee, 132 met Severe Maternal Morbidity (SMM) criteria defined by the American College of Obstetricians and Gynecologists (ACOG). Fewer SI reports were filed on Asian patients and those who declined to provide race or ethnicity (observed 4.3% versus expected 5.5%, p=0.0088 and 2.9% versus expected 1%, p<0.0001, respectively). For cases reviewed by the departmental PSQI committee and for those which met SMM criteria, there was no significant difference in race/ethnicity distribution.

Conclusions

There was a disparity between fewer safety events filed for Asian patients and those not reporting race/ethnicity. It was reassuring that our process did not identify other racial/ethnic disparities. However, given the widespread systemic inequities in healthcare, further evaluation of our PSQI process, and PSQI processes beyond our institution, is needed.

## Introduction

The United States has one of the highest maternal mortality and Severe Maternal Morbidity (SMM) rates compared to other developed countries affecting 17 people per 100,000 births and up to two percent of all people giving birth, respectively [1-6]. Maternal death is defined as pregnancy-related death while pregnant or within one year of the end of pregnancy from any cause related to or aggravated by the pregnancy [1]. While there is no uniform definition of SMM, it encompasses any unexpected or near-miss outcome that puts pregnant people at risk of dying, such as eclampsia, cardiovascular events, hemorrhage, sepsis, and organ failure [5-6]. It is well established that birthing people of color, particularly Black people, experience a three- to four-fold higher risk of maternal mortality and two- to three times higher risk of SMM than White people [1,6-9]. Pregnancy-related mortality is also higher among Native Americans/Native Alaskans, Asians/Pacific Islanders, and for specific subgroups of Latina women [10]. These significant disparities persist even after adjusting for individual-level and socioeconomic factors [6, 7, 10]. Further, the gap in disparate obstetric outcomes has widened over the past decades [7-9].

Most maternal deaths are deemed preventable and are thought to be related to delay in treatment or diagnosis, lack of clear communication, or deficiency of institutional policies and procedures [10]. Structural racism has been increasingly identified as a cause for disparities in health outcomes [7,11-13]. While there is a lack of studies evaluating structural racism and its effect on obstetric outcomes, data suggest that it has a detrimental effect on maternal outcomes [7,11]. People of color are more likely to have limited access to prenatal and postpartum care and are more likely to deliver in lower-quality hospitals [7-10].

There is an urgent call for action to decrease maternal morbidity and mortality in the United States and to eliminate the racial gap in outcomes by increasing provider education, addressing implicit and explicit bias, standardization and implementing high-quality care bundles, increasing community engagement, and implementing policy - level changes to address social determinants of health and systemic racism [6,10-16]. The Alliance for Innovation of Maternal Health (AIM) calls for the consideration of equity in every safety bundle at all levels of implementation [16]. 

Despite the plethora of evidence describing racial and ethnic disparities in obstetric care and maternal outcomes [1-6,10], little has been published evaluating potential inequities in departmental Patient Safety and Quality Improvement (PSQI) reporting and review processes. The study aims to describe the distribution of patient self-reported race or ethnicity for safety events filed by the care team members and for cases that the departmental multidisciplinary PSQI committee reviewed at recurrent monthly meetings at a single safety net teaching hospital. We hypothesized that the percent distribution of race or ethnicity of observed and expected cases is similar, signifying proportional representation in our departmental PSQI reporting and review process. 

## Materials and methods

We performed a cross-sectional study that included all reported safety events filed on obstetric and gynecologic patients and all cases reviewed at a monthly PSQI multidisciplinary departmental meeting from May 2016 to December 2021. The study was conducted at a large volume, safety-net teaching hospital with an average of 3300 deliveries per year and 850 inpatient and outpatient gynecologic procedures per year. Per institutional practice, cases with adverse outcomes, near misses, communication problems, technical issues, medication errors, or any other concerns the care team raises are filed in a centralized Safety Intelligence (SI) system. SI events are then reviewed by various stakeholders, including nursing and physician leadership, residency program leadership, hospital administrators in the department of Patient Safety and Quality, and other services involved in patient care as deemed pertinent. Selected SI events, including adverse outcomes, misses or near misses as required by regulatory agencies, those that meet severe maternal morbidity (SMM) criteria as defined by the American College of Obstetricians and Gynecologists (ACOG) or cases that provide an opportunity for process improvement are reviewed by the department of OB/GYN PSQI committee. The committee is multidisciplinary, with representation from nursing, OB/GYN physicians, certified nurse midwives (CNM), Family Medicine, Neonatology, Anesthesia, the department of Patient Safety and Quality, and Risk Management, and meets at recurrent peer review protected monthly meetings. To promote transparency and staff engagement in the departmental PSQI reporting and review process, the committee also reviews cases as requested by healthcare team members, even if the case does not meet the review mentioned above criteria. In addition, the departmental PSQI meetings are open to any healthcare team members who wish to attend. 

The primary outcome of our study is the distribution of patients' self-reported race or ethnicity for all safety events. Of note, the latter information is not included in SI reports and is only apparent once the case is selected for review by the PSQI committee. Patient self-reported race or ethnicity was abstracted from the electronic medical record for all SI events and cases reviewed by the departmental PSQI committee. Secondary outcomes include the distribution of maternal race or ethnicity in SMM cases defined as transfusion of 4 units or more of packed red blood cells, unplanned peripartum hysterectomy, unplanned admission to the intensive care unit, maternal cardiovascular event, death, unexpected return to the operating room, retained foreign body, and sepsis. We compared observed versus expected distributions based on historical institutional data where 61% of patients identify as Latina/Hispanic, 14.5% as Non-Hispanic Black, 17% as Non-Hispanic White, 5.5% as Asian, 1% as others including Alaska Native, Pacific Island Native, Native Hawaiian, and those who declined to answer (1%). 

The Denver Health and Hospital Authority Quality Improvement Committee exempted the study, authorized by the Colorado Institutional Review Board.

## Results

**Figure 1:**
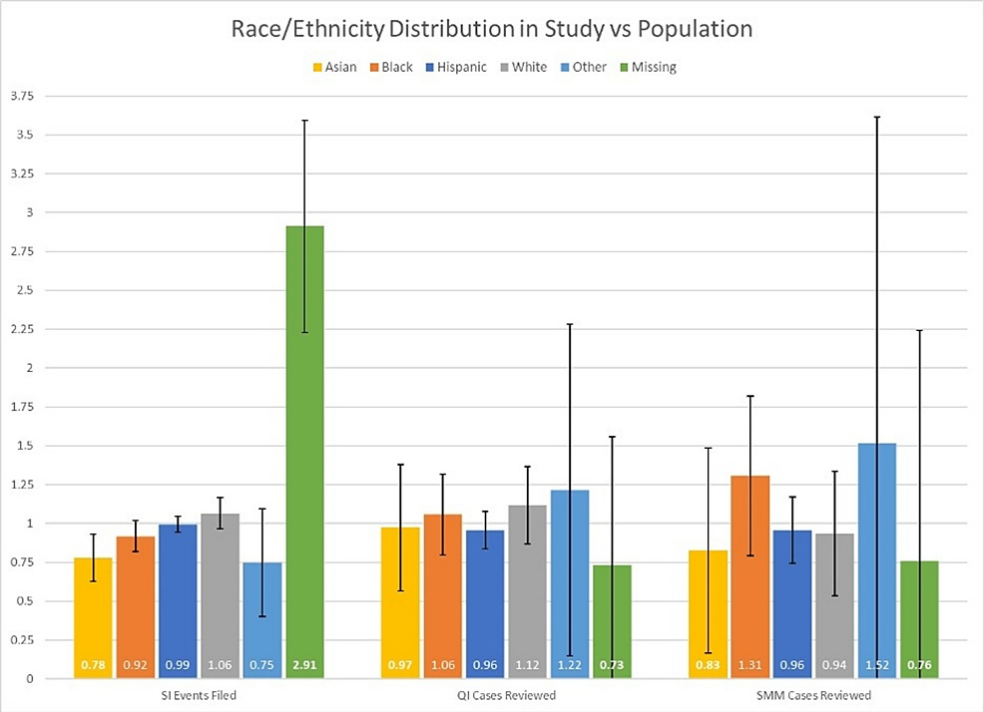
Observed versus expected distribution of patient self-reported race or ethnicity in Patient Safety and Quality Improvement processes at a single safety net academic institution.

A total of 2405 SI events were filed on obstetric and gynecologic patients. Of those, 411 cases were reviewed during monthly multidisciplinary departmental PSQI meetings, and 132 met SMM criteria. The distribution of observed and expected race/ethnicity for SI events, PSQI review cases, and SMM cases is presented in Table 1. For SI events, based on the expected distribution, significantly fewer reports were filed for patients who identified as Asian and for those who declined to answer (observed 4.3% versus expected 5.5%, p=0.0088, and observed 2.9% versus expected 1%, p<0.0001, respectively). There was no significant difference between the observed and expected distribution for all other races and ethnicities. Similarly, there was no difference in the observed and expected distribution of race and ethnicity for cases reviewed by the departmental PSQI committee and for the subset of cases that met SMM criteria. The ratio of observed versus expected race/ethnicity distribution is further presented in Figure 1.

**Table 1:** Observed and expected distribution of patient self-reported race or ethnicity in Patient Safety and Quality Improvement cases at a single safety net academic institution. ^a^ Self-reported by patients as recorded in the electronic medical record ^b^ Based on historical institutional data 2017 – 2019 ^c^ Includes Alaska Native, Pacific Island Native, Native Hawaiian, or patients who declined to answer PSQI – Patient Safety and Quality Improvement; SMM – Severe Maternal Morbidity

Race/Ethnicity ^a^		Observed, N (%)	Expected ^b ^ %	Observed/Expected	P value
Safety Intelligence Events (N=2405)	Asian	103 (4.3%)	5.5%	0.78	0.0088
	Black	320 (13.3%)	14.5%	0.92	0.0962
	Hispanic/Latina	1459 (60.7%)	61%	0.99	0.0962
	White	435 (18.1%)	17%	1.06	0.1557
	Other ^c^	18 (0.2%)	1%	0.75	0.2150
	Missing	70 (2.9%)	1%	2.91	<0.0001
Departmental PSQI Review Events (N=411)	Asian	22 (5.4%)	5.5%	0.97	0.8959
	Black	63 (15.3%)	14.5%	1.06	0.6334
	Hispanic/Latina	240 (58.4%)	61%	0.96	0.2788
	White	78 (19.0%)	17%	1.12	0.2857
	Other ^c^	5 (1.2%)	1%	1.22	0.6591
	Missing	3 (0.7%)	1%	0.73	0.5821
SMM Events (N=132)	Asian	6 (4.5%)	5.5%	0.83	0.6305
	Black	25 (18.9)	14.5%	1.31	0.1475
	Hispanic/Latina	77 (58.3%)	61%	0.96	0.5299
	White	21 (15.9%)	17%	0.94	0.7386
	Other ^c^	2 (1.5%)	1%	1.52	0.5519
	Missing	1 (0.01%)	1%	0.76	0.7795

## Discussion

Principal findings

In this cross-sectional study, we found significantly fewer SI events filed by the clinical care team members on patients who identify as Asians (p=0.0088) and those who had declined to answer (p<0.0001). There were no significant differences in the observed and expected distribution of race and ethnicity for cases reviewed by the departmental PSQI committee and those that met SMM criteria.

Clinical and Research Implications

Numerous studies have shown significant racial and ethnic disparities in maternal outcomes and SMM. Recently published data consistently identified higher SMM rates in Black, Latina, and other minority patients [2-4]. Our study was not designed to compare SMM in different patient populations; however, about reporting and review of notable safety events, including SMM, there was no significant difference in the distribution of race or ethnicity in the number of reported and reviewed cases compared to expected distribution based on historical institutional data. 

Strengths and Limitations

The study has several notable strengths. This first report investigates racial and ethnic disparities in a departmental PSQI reporting and review process. Further, the study was comprehensive, including all filed safety events for obstetric and gynecologic patients and all cases the PSQI committee reviewed in the department during the study period. For cases where there needed to be more data, an additional manual chart review was performed to ensure limited missing data. Only 70 cases (2.9%) had missing race/ethnicity data, which notably included patients who declined to answer. 

The study also has several notable limitations. The study's cross-sectional design is only hypothesis-generating and limits the ability to draw definitive conclusions. Further, the study design does not exclude the possibility of selection and reporting bias. While the criteria for filing a SI event are clearly delineated and readily available to staff, and the reporting process involves a minimal time commitment, it is possible that events that involved patients of color were underreported, thus skewing our results toward the null hypothesis. In addition, given that the study was conducted at a single institution, the findings may need more external validity and generalizability based on our patient population.

Further, the study may be underpowered to detect significant disparities in race or ethnicity distribution of cases reviewed by the PSQI committee and among those which meet SMM criteria. Lastly, the ACOG Obstetric Care Consensus [5] delineating criteria for SMM was published in September 2016. SMM cases were under-reported by the departmental PSQI committee while the criteria were being adopted and integrated into the review process.

## Conclusions

While there is a plethora of evidence describing racial and ethnic disparities in obstetric care and outcomes, little has been published evaluating potential inequities in departmental Patient Safety and Quality Improvement (PSQI) processes. In this cross-sectional study performed at a single safety net academic institution, we found a disparity between fewer safety events filed for Asian patients and those who did not report race or ethnicity. It was reassuring that our quality improvement reporting and review process did not identify other racial and ethnic disparities. However, given the widespread systemic inequities in healthcare, further evaluation of our PSQI process, and PSQI processes beyond our institution, is needed.

## References

[1] (2023). Centers for Disease Control and Prevention. Reproductive Health: Pregnancy Mortality Surveillance System. https://www.cdc.gov/reproductivehealth/maternal-mortality/pregnancy-mortality-surveillance-system.htm#race-ethnicity.

[2] Sutton MY, Anachebe NF, Lee R, Skanes H (2021). Racial and ethnic disparities in reproductive health services and outcomes, 2020. Obstet Gynecol.

[3] Howell EA, Egorova NN, Janevic T, Brodman M, Balbierz A, Zeitlin J, Hebert PL (2020). Race and ethnicity, medical insurance and within-hospital severe maternal morbidity disparities. Obstet Gynecol.

[4] Wang E, Glazer KB, Sofaer S, Balbierz A, Howell EA (2021). Racial and ethnic disparities in severe maternal morbidity: a qualitative study of women’s experience of peripartum care. Womens Health Issues.

[5] Kilpatrick SK, Ecker JL (2016). Severe maternal morbidity: screening and review. Am J Obstet Gynecol.

[6] Carmichael SL, Abrams B, El Ayadi A (2022). Ways forward in preventing severe maternal morbidity and maternal health inequities: conceptual frameworks, definitions, and data, from a population health perspective. Wom Health Issues.

[7] Hailu EM, Maddali SR, Snowden JM, Carmichael SL, Mujahid MS (2022). Structural racism and adverse maternal health outcomes: a systematic review. Health Place.

[8] Creanga AA, Bateman BT, Kuklina EV, Callaghan WM (2014). Racial and ethnic disparities in severe maternal morbidity: a multistate analysis, 2008-2010. Am J Obstet Gynecol.

[9] Creanga AA, Bateman BT, Mhyre JM, Kuklina E, Shilkrut A, Callaghan WM (2014). Performance of racial and ethnic minority-serving hospitals on delivery-related indicators. Am J Obstet Gynecol.

[10] Howell EA (2018). Reducing disparities in severe maternal morbidity and mortality. Clin Obstet Gynecol.

[11] Crear-Perry J, Correa-de-Araujo R, Lewis Johnson T, McLemore MR, Neilson E, Wallace M (2021). Social and structural determinants of health inequities in maternal health. J Womens Health (Larchmt).

[12] Taylor JK (2020). Structural racism and maternal health among Black women. J Law Med Ethics.

[13] Minehart RD, Bryant AS, Jackson J, Daly JL (2021). Racial/ethnic inequities in pregnancy-related morbidity and mortality. Obstet Gynecol Clin North Am.

[14] Jain JA, Temming LA, D'Alton ME (2018). SMFM special report: putting the “M” back in MFM: addressing education about disparities in maternal outcomes and care. Am J Obstet Gynecol.

[15] Lee A, Padilla C (2022). Causes of health inequities. Curr Opin Anaesthesiol.

[16] (2023). Council on Patient Safety in Women’s Health Care. https://saferbirth.org.

